# Comparative assessment of METastasis Reporting and Data System for Prostate Cancer versus Prostate Cancer Clinical Trials Working Group 3 Criteria in assessing treatment response in patients with metastatic castration-resistant prostate cancer

**DOI:** 10.1016/j.euros.2026.08.003

**Published:** 2026-08-20

**Authors:** Luca Russo, Ossian Longoria, Silvia Bottazzi, Nuria Porta, Katie Biscombe, Samuel J. Withey, Giacomo Avesani, Katarzyna Abramovicz, Matthew D. Blackledge, Luca D’Erme, Katie Russell-Rose, Tarek Taha, Adam Sharp, Alec Paschalis, Evis Sala, Dow-Mu Koh, Johann de Bono, Nina Tunariu

**Affiliations:** a The Royal Marsden NHS Foundation Trust, London, UK; b Fondazione Policlinico Universitario A. Gemelli IRCCS, Rome, Italy; c Institute of Cancer Research, London, UK; d Università Cattolica del Sacro Cuore, Rome, Italy

**Keywords:** Bone imaging, Metastatic castration-resistant prostate cancer, Whole-body magnetic resonance imaging, Response biomarkers

## Abstract

**Background:**

Unequivocal clinical progression without radiological progression according to Prostate Cancer Clinical Trials Working Group 3 (PCWG3) criteria is recognised in metastatic castration-resistant prostate cancer (mCRPC), reflecting limitations of bone scintigraphy in distinguishing an osteoblastic progression from an osteoblastic flare.

**Objective:**

To compare the METastasis Reporting and Data System for Prostate Cancer (MET-RADS-P) on whole-body magnetic resonance imaging (WBMRI) with PCWG3 imaging criteria, the current gold standard.

**Design, setting, and participants:**

A retrospective cohort of 184 patients with mCRPC who underwent paired WBMRI and conventional imaging during therapy.

**Outcome measurements and statistical analysis:**

The primary endpoint was concordance (percentage agreement [%A]) between MET-RADS-P and PCWG3 for overall, bone-only, and soft-tissue-only responses. Secondary endpoints were radiologic progression-free survival (rPFS) by each system and overall survival (OS) according to early (≤12 wk) MET-RADS-P response.

**Results and limitations:**

We evaluated 467 paired assessments in 184 patients. Overall concordance between PCWG3 and MET-RADS-P was moderate (%A = 64.5%; 95% confidence interval [CI] = 59.8–68.7), and it was high in soft tissue (%A = 88.8%; 95% CI = 85.7–91.6) and limited in bone (%A = 44.4%; 95% CI = 39.8–48.9). MET-RADS-P labelled progression more often than PCWG3 overall (66.6% vs 39.7%) and in bone (61.2% vs 20.3%). Median rPFS was shorter by MET-RADS-P than by PCWG3 (2.7 vs 4.2 mo; *p* < 0.001). Early MET-RADS-P progression was significantly associated with worse OS (adjusted hazard ratio [HR] = 4.75; 95% CI = 2.1–10.7; *p* < 0.001). Limitations include retrospective single-centre design, trial-based expert-centre cohort, and limited follow-up.

**Conclusions:**

Concordance between PCWG3 and MET-RADS-P was moderate overall and limited in bone. Earlier MET-RADS-P–defined progression may identify aggressive disease and allow a prompt change in therapy before clinical deterioration limits subsequent treatment options. Prospective validation is warranted.

## Take Home Message

Prostate Cancer Clinical Trials Working Group 3 criteria based on conventional imaging (computed tomography and bone scanning) are not concordant with METastasis Reporting and Data System for Prostate Cancer (MET-RADS-P) criteria, based on whole-body magnetic resonance imaging (WBMRI) with diffusion-weighted imaging, mainly because of the underdetection of bone disease progression. Prospective trials incorporating MET-RADS-P are urgently needed to validate these findings, which may lead to increased access to WBMRI and improved patient outcomes.

ADVANCING PRACTICE **What does this study add?**

Prostate Cancer Clinical Trials Working Group 3 (PCWG3) criteria are the standardised response assessment criteria for metastatic castration-resistant prostate cancer but have important limitations, especially in the assessment of bone metastases response to treatment. The METastasis Reporting and Data System for Prostate Cancer (MET-RADS-P) may improve upon PCWG3 in these respects, but these two sets of criteria have not been compared directly before. This study suggests that criteria that incorporate whole-body magnetic resonance imaging and diffusion-weighted imaging, such as MET-RADS-P, may improve tumour response assessments and, if these findings are prospectively validated, may represent powerful prognosticators of clinical outcomes.

**Clinical Relevance**

MET-RADS-P may identify progression in mCRPC earlier than conventional PCWG3 criteria, particularly in bone, where osteoblastic flare can confound true disease progression. Earlier and accurate identification of progression may allow clinicians to reassess treatment efficacy and modify systemic therapy before clinical deterioration limits subsequent treatment options. Prospective validation is needed to determine whether incorporating MET-RADS-P into treatment decision-making can improve the timing and sequencing of subsequent therapies and ultimately patient outcomes. Associate Editor: Roderick C.N. van den Bergh.

**Patient Summary**

Conventional scans and the Prostate Cancer Clinical Trials Working Group 3 (PCWG3) criteria used to assess tumour response in advanced prostate cancer facilitate therapeutic decisions but lack detail on bone metastases. In this work, we studied the concordance between PCWG3 (the current gold standard) and the newer METastasis Reporting and Data System for Prostate Cancer criteria, which incorporate whole-body magnetic resonance imaging (MRI) and demonstrated that concordance is limited in bone. These results suggest that further work is warranted to validate and incorporate MRI as an imaging tool able to detect earlier progression that may lead to a therapy switch and improved clinical outcomes.

## 1 Introduction

Metastatic castration-resistant prostate cancer (mCRPC) is a lethal disease. New therapies have been reported to improve median overall survival (OS) in mCRPC from 26 mo (one line of systemic treatment) to 52 mo (two or more lines of systemic treatment) [1].

Treatment response in mCRPC is currently assessed using prostate-specific antigen (PSA) and conventional imaging—computed tomography (CT) plus bone scintigraphy (BS)—as recommended by the Prostate Cancer Clinical Trials Working Group 3 (PCWG3) [2]. PSA is an inadequate surrogate for OS and not uncommonly discordant with radiological response [3], [4]; up to one quarter of patients progress radiologically despite stable or falling PSA, reflecting lineage plasticity [5], [6], [7]. CT has limited accuracy for bone marrow metastases, particularly in sclerotic lesions, and lacks established response criteria [8]. BS reflects osteoblastic activity rather than tumour burden and is prone to flare and false-negative results [9]; therefore, PCWG3 relies on progression-only bone criteria with a confirmatory scan, which can delay treatment decisions. Unequivocal clinical progression in the absence of radiological progression has been reported in 38–68% of the patients with mCRPC [10], [11]. Prostate-specific membrane antigen positron emission tomography (PSMA-PET) guides selection for PSMA-targeted therapy, but most mCRPC tumours show heterogeneous PSMA expression and almost half harbour PSMA-negative lesions [12], [13], [14]. Because three-quarters of patients with mCRPC have bone disease [15], optimal bone-response assessment remains a major unmet need.

Whole-body magnetic resonance imaging (WBMRI) with diffusion-weighted imaging (DWI) offers comprehensive, radiation-free assessment of both soft-tissue and bone lesions [16] and prompt detection of skeletal-related complications such as malignant spinal cord compression. METastasis Reporting and Data System for Prostate Cancer (MET-RADS-P) provides a standardised approach to WBMRI acquisition, interpretation, and reporting, allowing structured assessment of response [17], [18], [19], [20], [21], [22]. Magnetic resonance imaging (MRI)–based disease classification correlates with viable tumour cell presence on bone marrow biopsy [23]. Therefore, WBMRI may provide clinically relevant information beyond conventional imaging, particularly in patients in whom bone-predominant disease, mixed response patterns, or discordance between PSA and imaging complicate treatment monitoring. MET-RADS-P has not yet been integrated into clinical trial protocols, and evidence supporting its clinical utility remains limited [24]. We compared MET-RADS-P with PCWG3 criteria (the current gold standard) for response assessment in a single-centre mCRPC cohort and explored prognostic associations between MET-RADS-P categories and survival.

## 2 Patients and methods

### 2.1 Patients

We retrospectively analysed consecutive patients with mCRPC treated in PCWG3-compliant clinical trials at a tertiary cancer centre (The Royal Marsden NHS Foundation Trust, London, UK) between January 2013 and February 2024. Institutional Ethics Committee approval was obtained (reference number 21/LO/0605). WBMRI was routinely used for response assessment; trial protocols mandated conventional imaging (CT and BS) every 9–12 wk, as per PCWG3, providing contemporaneous imaging pairs. Eligible patients had histologically confirmed prostate cancer, documented progression to mCRPC, paired baseline WBMRI and conventional imaging within 30 d before starting treatment, and one or more on-treatment paired assessments. Patients could contribute multiple treatment lines if new paired baselines and at least one paired follow-up were available. Treatment discontinuation decisions were made by the clinical team according to PCWG3 criteria. MET-RADS-P assessments were performed retrospectively and did not influence management.

### 2.2 CT and BS

CT of the chest, abdomen, and pelvis with intravenous iodinated contrast was performed unless contraindicated. BS used technetium-99m–labelled methylene diphosphonate with whole-body planar acquisition.

### 2.3 WBMRI

WBMRI examinations were performed on 1.5 Tesla scanners (Siemens Healthineers, Erlangen, Germany) using MET-RADS-P-compliant protocols that included standard T1-weighted and T2-weighted anatomical sequences and DWI.

### 2.4 Imaging analysis

Two radiologists with 2 and 7 yr of experience in oncological imaging reviewed all imaging in consensus, with discrepancies resolved by a third radiologist with 20 yr of experience. For each patient and line of treatment, baseline and follow-up CT and BS studies were grouped and assessed during the same reading session according to PCWG3 criteria. If CT was unavailable, morphological sequences from WBMRI were used to evaluate soft-tissue metastases following RECIST version 1.1 [25]. Subsequently, WBMRI studies from corresponding timepoints were separately assessed according to the MET-RADS-P criteria.

### 2.5 Response assessment

Soft-tissue metastases (nodal and visceral) were evaluated by both systems using RECIST version 1.1 [25], generating complete response (CR), partial response (PR), stable disease (SD), and progressive disease (PD).

Under PCWG3, bone metastases were assessed by BS: ≥2 new lesions versus baseline or a prior follow-up were classified as unconfirmed PD (PD-U) and confirmed 6–12 wk later by the “2 + 2” or “2 + 0” rules, depending on the timing relative to the 12-wk flare period. The absence of new lesions, irrespective of tracer uptake, was categorised as non-PD; PCWG3 does not define response categories for bone disease. Under MET-RADS-P, bone disease was assessed on multiparametric WBMRI combining anatomical sequences with DWI. Response assessment categories (RACs) were assigned per MET-RADS-P recommendations (Supplementary Table 1): RAC 1–2 (responding), RAC 3 (stable), and RAC 4–5 (progressing).

### 2.6 Statistical analyses

Continuous variables were summarised as medians and 25th and 75th percentiles (interquartile range [IQR] = Q1–Q3) and categorical variables as frequencies and percentages. Concordance between systems was assessed for overall disease, bone only, and soft tissue only (RECIST version 1.1) at each contemporaneous timepoint. For overall disease, RAC 1–2 corresponded to CR-PR according to PCWG3, RAC 3 to SD, and RAC 4–5 to PD or PD-U. For bone-only disease, RAC 3 corresponded to non-PD and RAC 4–5 to PD or PD-U. For soft-tissue-only disease, the same RECIST 1.1 categories (CR-PR, SD, and PD) were directly compared. Percentage agreement (%A) with 95% exact confidence intervals (CIs) described raw concordance between systems across all paired scans, assuming independence of instances within the same patient on the same treatment. To account for the correlated structure of the data, a cumulative logit mixed model for each ordinal score was implemented. The models included system (MET-RADS-P and PCWG3) and line of treatment (first, second, and third) as fixed effects. Weeks on treatment were modelled as a continuous fixed factor via a smoothing spline. A random intercept for the patient accounted for within-patient correlation across repeated scans. The estimation of the model was done under a Bayesian framework using the R brms package [26]. From this model, the latent intraclass correlation coefficient (ICC) and probability of agreement between methods, together with 95% credible intervals, were estimated from the posterior distribution [27].

Radiologic progression-free survival (rPFS) for each imaging system was measured from pretreatment imaging to progression by that system or death for each patient and line of treatment. Patients without a progression event were censored at the last radiological assessment for the corresponding line of treatment. Kaplan-Meier curves for both systems were compared with paired log-rank tests; multiple lines of treatment within the same patient were considered to be independent instances for this analysis.

We explored the prognostic value of early MET-RADS-P response on OS among those patients who had a MET-RADS-P assessment within 12 wk of starting treatment. OS was measured from baseline (first line at our centre) to death because of any cause. If death was not recorded during the reporting period, patients were censored on the last date they were known to be alive. Landmark analysis (≤12 wk) was performed using Kaplan-Meier and Cox models, both univariable and multivariable. The multivariable model adjusted for treatment and a prespecified set of established prognostic factors, entered together without variable selection (Supplementary Methods). Median follow-up for time-to-event endpoints was computed using the reverse Kaplan-Meier method. A two-sided *p* < 0.05 was considered significant. Analyses were conducted using Stata (version 17; StataCorp LLC, College Station, TX, USA) and R (version 4.5.3; R Foundation for Statistical Computing, Vienna, Austria).

## 3 Results

### 3.1 Study population

**Fig. 1:**
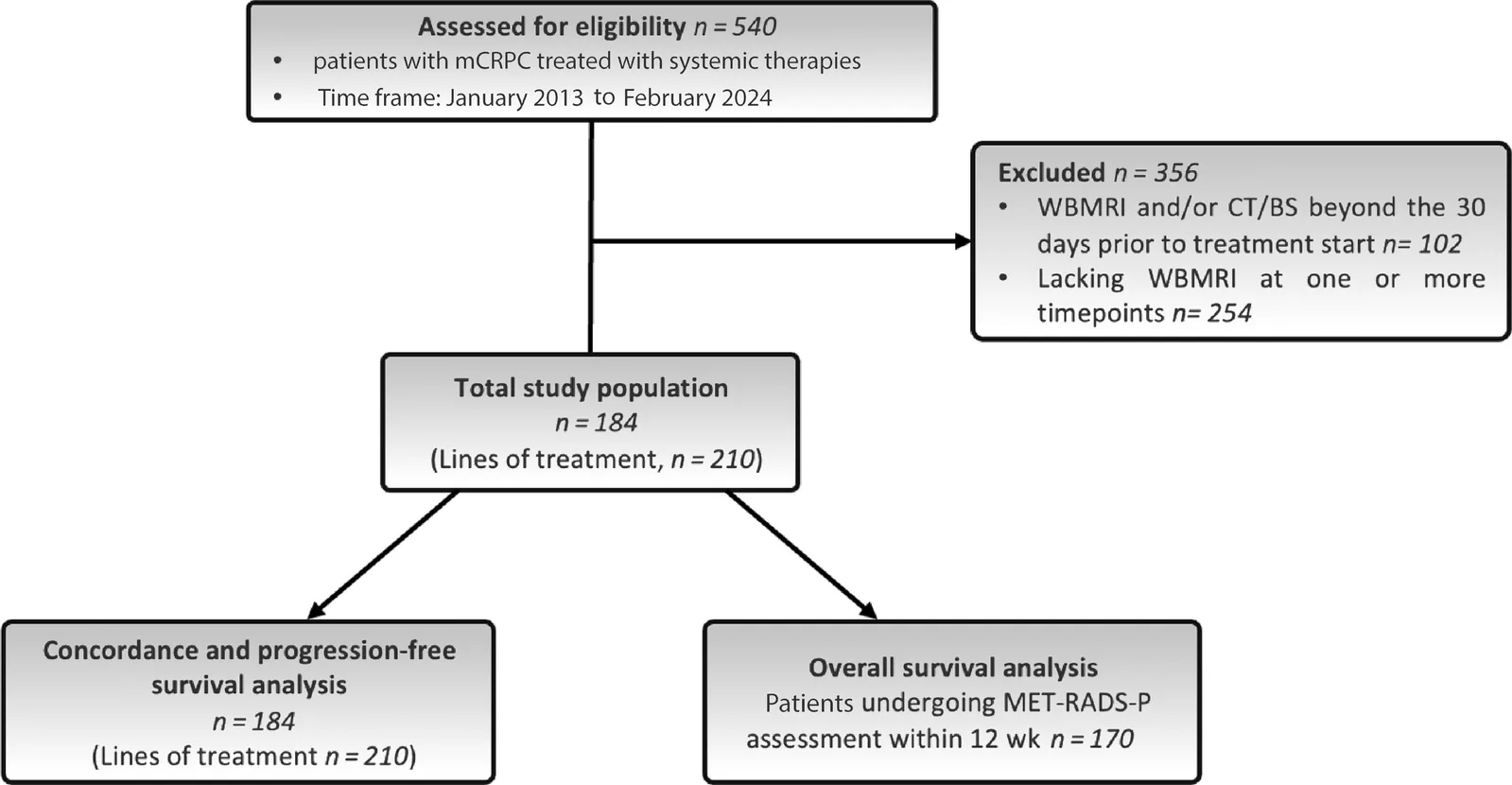
Flow diagram of the study population. BS = bone scintigraphy; CT = computed tomography; mCRPC = metastatic castration-resistant prostate cancer; MET-RADS-P = METastasis Reporting and Data System for Prostate Cancer; WBMRI = whole-body magnetic resonance imaging.

Overall, we identified 184 unique patients with mCRPC with paired baseline and follow-up imaging (Fig. 1). At baseline (first documented treatment at our centre), median age was 68 yr (IQR = 63–72), and median PSA was 95.4 ng/ml (IQR = 26.6–339.5). Metastases distribution included bone (*n* = 171, 92.9%), nodal (*n* = 84, 46%), and visceral involvement (*n* = 44, 24%). Baseline characteristics are detailed in Table 1. We included 210 lines of treatment for these 184 patients, with 23 patients having more than one line of treatment. Details of the systemic therapies are summarised in Supplementary Table 2.

**Table 1:** Baseline characteristics of the study population (N = 184)

**Characteristic**	**Median**	**IQR**
Age (yr)	68	63–72
Prostate-specific antigen (ng/ml)	95.4	26.6–339.5
Haemoglobin (g/l)	118	108–128
Platelets (×10^9^^/^l)	247.5	200.5–308.5
Alkaline phosphatase (IU/l)	125	87–207
Albumin (g/l)	41	37–44
Lactate dehydrogenase (IU/l)	201	169–251
	***n***	**%**
**Gleason score**		
≤7	44	23.9
8	32	17.4
9 or 10	85	46.2
NA	23	12.5
**ECOG performance status**		
0	24	13.0
1	154	83.7
2	6	3.3
**Use of opiates**		
No	120	65.2
Yes	64	34.8
**Nodal disease**		
No	100	54.4
Yes	84	45.6
**Bone disease**		
No	13	7.1
Yes	171	92.9
**Visceral disease**		
No	140	76.1
Yes	44	23.9
**Prior lines of treatment**		
≤1	29	15.8
2	51	27.7
3	64	34.8
≥4	40	21.7

ECOG = Eastern Cooperative Oncology Group; IQR = interquartile range; NA = not applicable.

### 3.2 Imaging analysis and concordance

Across 210 treatment lines, 677 imaging timepoints were available (210 baselines; 467 follow-ups). The median number of scans per patient and line of treatment was 2 (IQR = 2–4) CT scans, 3 (IQR = 2–4) bone scans, and 3 (IQR = 2–4) WBMRI scans. Only 10% of the patients had more than five scans per line of treatment.

**Fig. 2:**
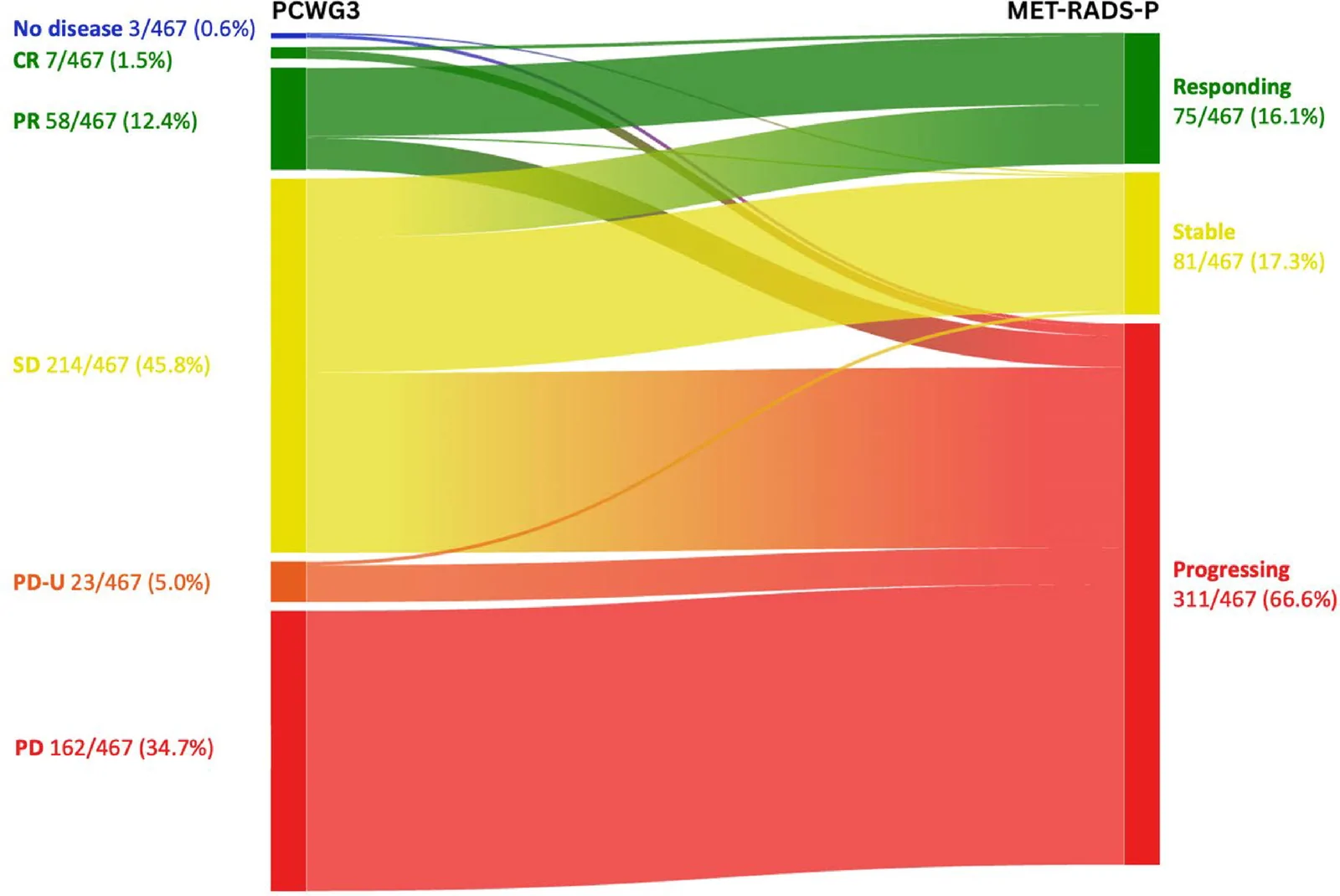
Sankey diagram illustrating the overall assessment of treatment response based on PCWG3 and MET-RADS-P criteria. The flow of patient classifications from PCWG3 (left) to MET-RADS-P (right) shows how patients were reclassified between the two systems. Patients classified as having a CR or PR by PCWG3 were mainly concordant with the responding category in MET-RADS-P, whereas most of those classified as having SD by PCWG3 were considered either stable or progressing according to MET-RADS-P. CR = complete response; MET-RADS-P = METastasis Reporting and Data System for Prostate Cancer; PCWG3 = prostate cancer clinical trials working group 3; PD = progressive disease; PD-U = unconfirmed progressive disease; PR = partial response; SD = stable disease.

Overall (*n* = 467), moderate concordance (301/467; %A = 65% [95% CI = 60–69]; weighted kappa coefficient (wκ) = 0.48 [95% CI = 0.40–0.53]) between MET-RADS-P and PCWG3 criteria was noted. MET-RADS-P classified more cases as progression (RAC 4–5: 67%) than PCWG3 (PD + PD-U: 40%). Of the 311 cases classified as progressive by MET-RADS-P, 103 (33%) were SD by PCWG3 (Fig. 2 and Supplementary Table 3).

**Fig. 3:**
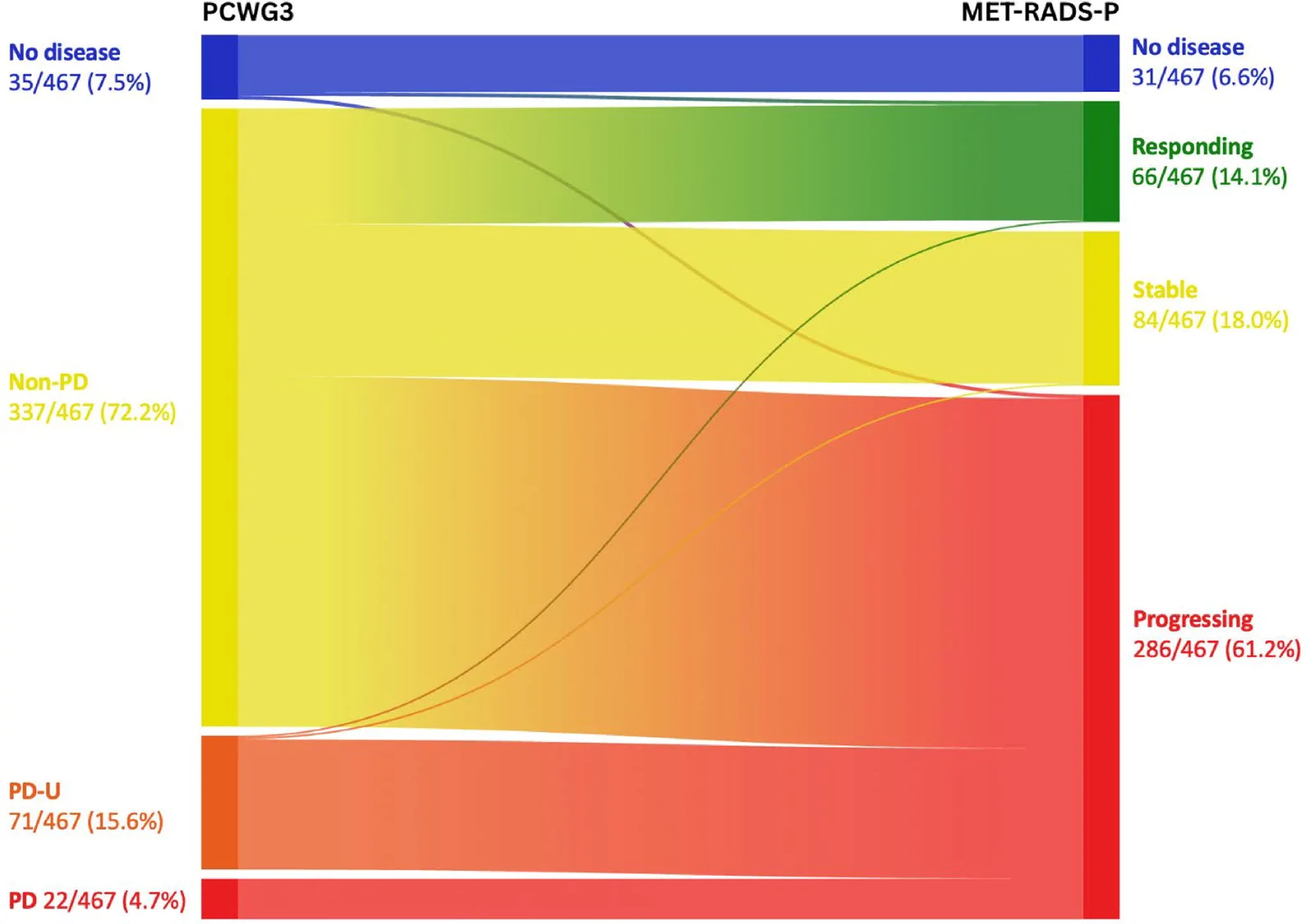
Sankey diagram illustrating the comparison of bone-only assessments between PCWG3 and MET-RADS-P criteria. PCWG3 (left) classifies patients as having no disease, non-PD, PD-U, or PD, whereas MET-RADS-P (right) categorises them as having no disease or responding, stable, or progressing disease. Discrepancies between the two systems are most notable in the non-PD group, in which a significant proportion of patients were reclassified as having progressing disease by MET-RADS-P. The diagram demonstrates that MET-RADS-P identifies more patients with PD (286 cases) than PCWG3 (95 cases). MET-RADS-P = METastasis Reporting and Data System for Prostate Cancer; PCWG3 = prostate cancer clinical trials working group 3; PD = progressive disease; PD-U = unconfirmed progressive disease.

For bone-only assessments (*n* = 467), concordance was limited (207/467; %A = 44% [95% CI = 40–49]; wκ = 0.40 [95% CI = 0.34–0.46]). MET-RADS-P identified progressive or likely PD (RAC 4–5) in 61% of the scans, whereas PCWG3 criteria classified only 20% (PD + PD-U) as progressive. Notably, of the 337 patients without PD according to PCWG3, 19% (63/337) were deemed to have responding disease, 25% (83/337) SD, and 57% (191/337) progressing disease according to MET-RADS-P (Fig. 3 and Supplementary Table 4). For the PD-U assessments, in which a confirmatory BS was not obtained (49/73), this was because of treatment discontinuation attributable to PSA and clinical progression in 12% (9/73) or soft-tissue RECIST progression in 55% of the cases (40/73).

Soft tissue-only concordance (*n* = 467) was high (415/467; %A = 89% [95% CI = 86–92], wκ = 0.85 [95% CI = 0.81–0.90]), reflecting shared RECIST 1.1 criteria (Supplementary Table 5 and Supplementary Fig. 1).

When adjusted for the correlated nature of the data, time and line of treatment, the latent ICCs for overall, bone and soft-tissue disease were 0.57, 0.73, and 0.85, respectively (Supplementary Table 6). The fitted mixed models also showed that there were systematic differences between the two systems, with MET-RADS-P providing larger scores than PCWG3, particularly for bone disease (Supplementary Table 6).

Illustrative case images of tumour response and progression detection in bone by WBMRI are shown in Supplementary Figs. 2 and 3, respectively.

### 3.3 PFS analysis

**Fig. 4:**
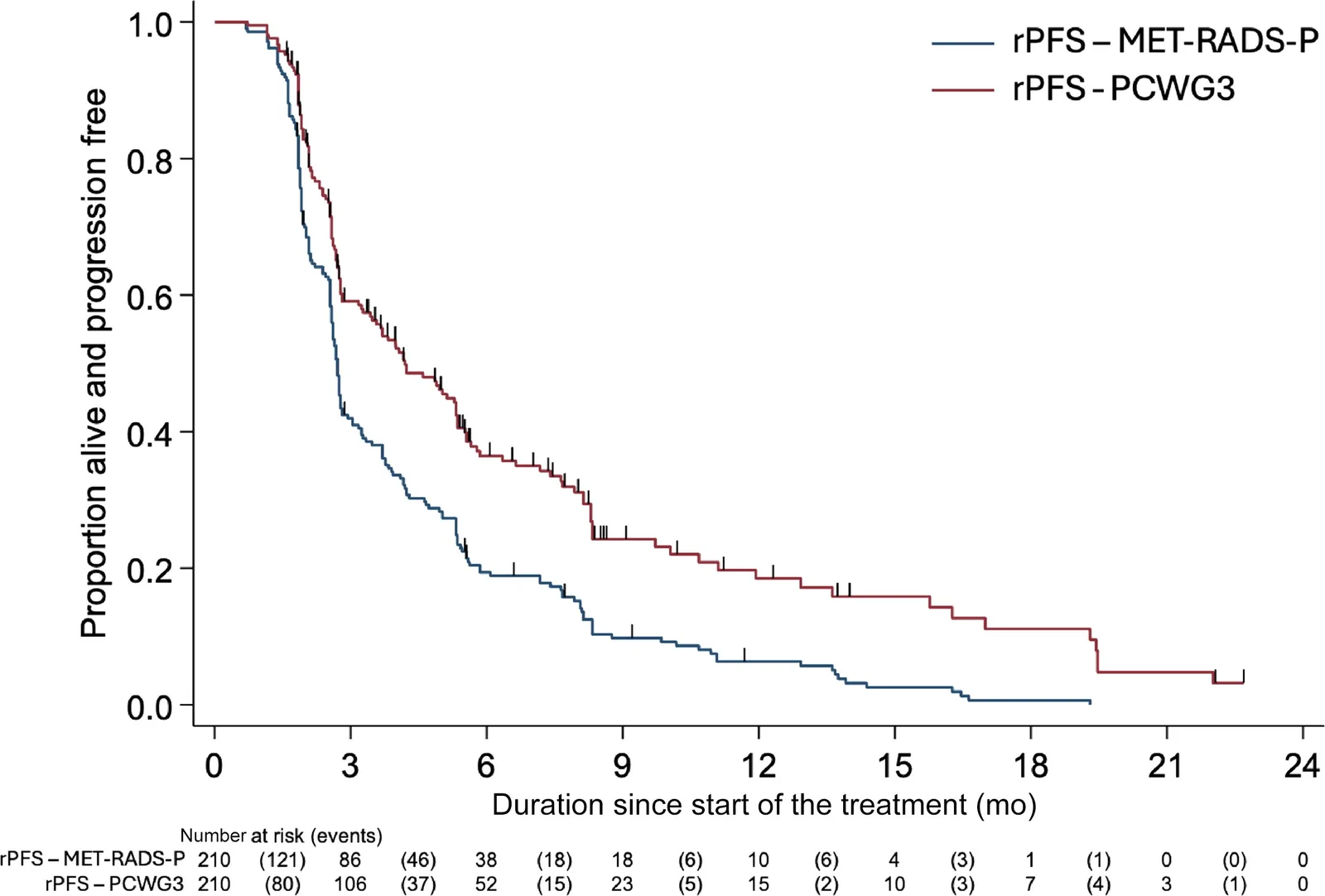
Kaplan-Meier curves comparing rPFS assessed using MET-RADS-P and PCWG3 criteria. The blue line represents rPFS according to MET-RADS-P, with a median of 2.7 mo (95% CI = 2.6–2.9), and the red line represents rPFS by PCWG3, with a median of 4.2 mo (95% CI = 3.4–5.3; paired log-rank *p* < 0.001). Note that the units of analysis are lines of treatment. CI = confidence interval; rPFS = radiologic progression-free survival; MET-RADS-P = METastasis Reporting and Data System for Prostate Cancer; PCWG3 = prostate cancer clinical trials working group 3.

Radiological progression by PCWG3 occurred in 147 of the 210 treatment lines (70%), with median rPFS of 4.2 mo (95% CI = 3.4–5.3; Fig. 4). Progression by MET-RADS-P occurred in 201 of the 210 lines of treatment (96%), with a shorter median rPFS (2.7 mo; 95% CI = 2.6–2.9; paired log-rank test *p* < 0.001).

### 3.4 OS analysis

**Fig. 5:**
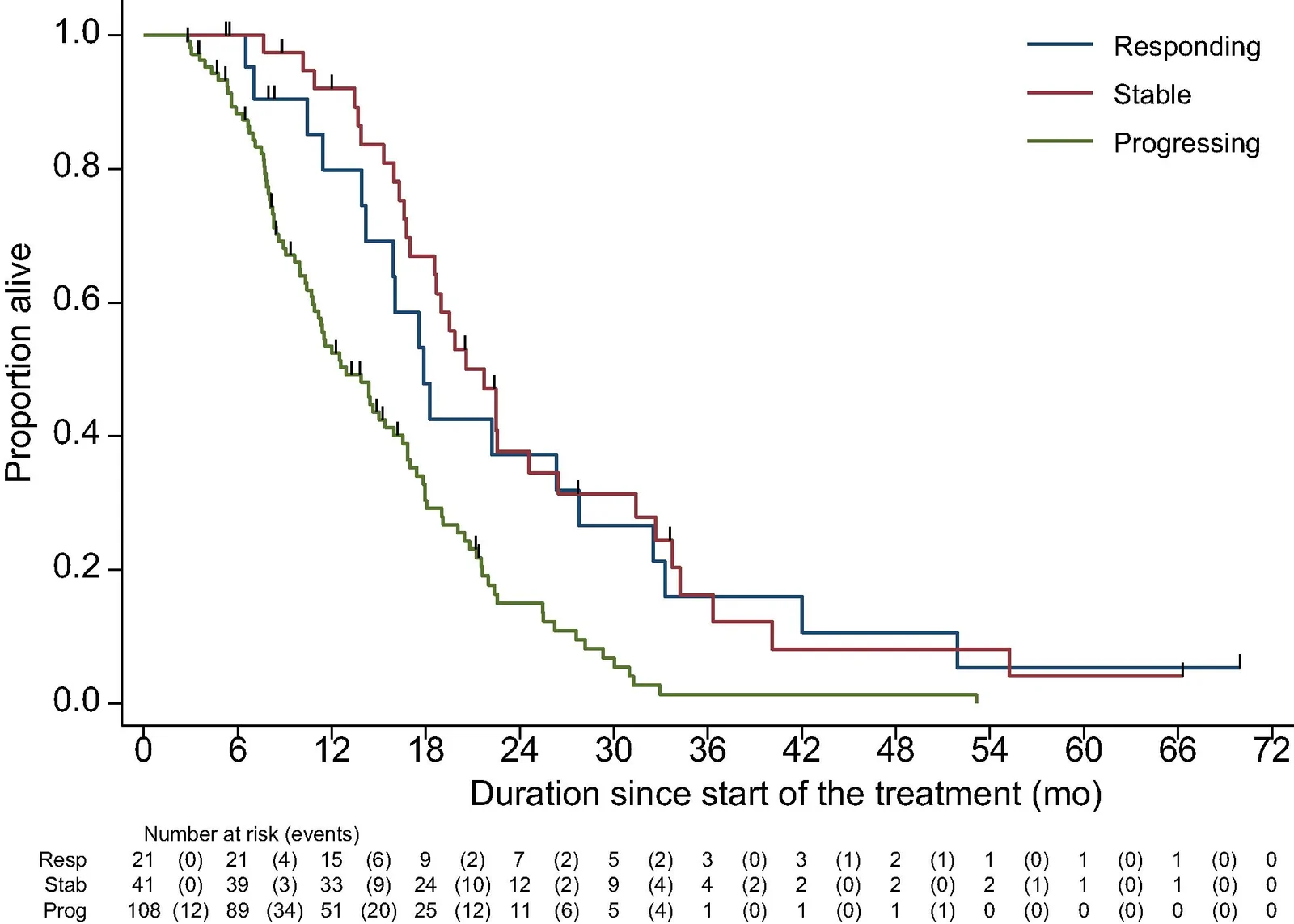
Kaplan-Meier curves comparing OS based on MET-RADS-P assessments performed within 12 wk of starting treatment. Patients with progressive disease had a median OS of 12.9 mo (95% CI = 10.8–16.0) compared with 21.7 mo (95% CI = 17.0–26.4) and 17.9 mo (95% CI = 13.9–27.8) for patients classified as having stable and responding disease, respectively (log-rank test *p* < 0.001). Note that the unit of analysis is the individual patient. CI = confidence interval; MET-RADS-P = METastasis Reporting and Data System for Prostate Cancer; OS = overall survival; Resp = Responding; Stab = Stable; Prog = Progressing.

We observed 138 deaths among 170 patients with an early MET-RADS-P (≤12 wk) assessment, with median follow-up for OS of 66 mo (Q1–Q3: 22–70). OS differed significantly for different MET-RADS-P categories (log-rank test *p* < 0.001): responding disease 17.9 mo (95% CI = 13.9–27.8), SD 21.7 mo (95% CI = 17.0–26.4), and PD 12.9 mo (95% CI = 10.8–16.0; Fig. 5). Early MET-RADS-P progression was independently associated with shorter OS on multivariable analysis (progressing vs responding: HR = 4.75; 95% CI = 2.1–10.7; *p* < 0.001), alongside age, opiate use, shorter time from diagnosis, and ≥3 prior lines (Supplementary Table 7).

## 4 Discussion

In this study, we compared WBMRI response assessment using MET-RADS-P with conventional PCWG3 criteria in patients with mCRPC. Concordance was moderate (65% of timepoints matched). Discrepancies were most evident in bone: MET-RADS-P classified 61% as progressive versus 20% by PCWG3.

Furthermore, rPFS was significantly shorter by MET-RADS-P than by PCWG3 (median 2.7 vs 4.2 mo), and WBMRI-defined progression within 12 wk of treatment initiation was associated with worse OS than WBMRI-defined stable or responding disease.

Our findings align with recent prospective studies. In the iPROMET trial, patients with responding disease (RAC 1–2) had longer times to progression than patients with nonresponding disease (RAC 3–5) [28]. Van Damme et al [29] reported PSA-WBMRI discordance in mCRPC, with approximately two-thirds of patients with a PSA response showing MET-RADS-P progression that was strongly associated with mortality and earlier initiation of subsequent therapy.

The PCWG3 framework is inherently limited in evaluating bone metastases because it cannot identify tumour response in bone metastases and requires the appearance of at least two new lesions followed by a confirmatory scan to establish progression. Such reliance on indirect osteoblastic activity and the confounding “flare” phenomenon introduces diagnostic latency [30]. By contrast, a flare phenomenon has not been described on diffusion-weighted WBMRI [31]; this further reduces the risk of misclassification when assessing on-treatment response in bone disease [16], [32].

MET-RADS-P captures biologically meaningful changes in tumour viability and MRI-derived biomarkers and has demonstrated a strong correlation with histopathological viability, even in sclerotic bone lesions [33].

PSMA-PET/CT has emerged as a sensitive molecular imaging tool, but its role in treatment response monitoring remains uncertain [34]. PSMA-PET Progression and Response Evaluation Criteria in PSMA Imaging (RECIP) 1.0 criteria have been proposed, incorporating new lesion detection and volumetric changes in PSMA-avid tumour burden [35], [36]. These metrics correlate with survival but remain limited in low- or absent-PSMA phenotypes [37], [38]. Dual-tracer PSMA/fluorodeoxyglucose PET addresses this, but cost, scanner time, and radiation constrain routine adoption [38]. In this context, MET-RADS-P may offer a complementary and reproducible approach [22].

WBMRI is a 45-min, contrast-free examination performed on standard 1.5-T scanners that detects lesions by cellularity and is unaffected by PSMA expression. It could replace CT, BS, and dedicated MRI spine/pelvis/prostate studies in a single visit and, by identifying progression earlier, reduce the duration of nonbeneficial therapy.

As with the Prostate Imaging Reporting and Data System (PI-RADS) for prostate cancer diagnosis, broader adoption of WBMRI will require expanded scanner capacity, protocol standardisation, and reader training. Cost-effectiveness and direct comparison with PSMA-PET/CT remain priorities for future investigation.

These findings should be interpreted within the current expert consensus on imaging in advanced prostate cancer. At the 2024 Advanced Prostate Cancer Consensus Conference, no consensus was reached on routinely substituting conventional imaging with WBMRI or PSMA-PET; both modalities were instead recommended as complementary tools when conventional imaging is discordant with PSA kinetics or clinically equivocal [39]. Our finding that MET-RADS-P reclassifies more than half of PCWG3 bone non-PD scans as progressive supports a targeted role for WBMRI, pending prospective validation.

This study has limitations. Its retrospective design (although using prospectively acquired clinical trial data) may introduce bias, and OS follow-up was relatively short. Patients could be included more than once if undergoing more than one treatment, but we assumed independence amongst their corresponding times to progression. Some treatments were nonstandard given the trial setting; contemporaneous imaging pairs partly mitigate this. The single tertiary-centre setting, standardised WBMRI protocols, and expert-reader interpretation may overestimate feasibility and reproducibility compared with routine practice. Imaging was assessed in consensus; independent interreader agreement before consensus was therefore not recorded. However, MET-RADS-P has shown excellent interobserver agreement, even between readers of differing expertise [18].

## 5 Conclusions

In conclusion, this study demonstrates that MET-RADS-P applied to WBMRI enables earlier detection of disease progression in mCRPC than PCWG3 criteria, particularly in metastatic bone disease, and that MET-RADS-P-defined progression may be associated with lower OS. Although MET-RADS-P is not implemented in clinical trials, our data support its potential utility as a more sensitive and standardised treatment response assessment. Detecting earlier radiological progression before clinical progression may expose patients with mCRPC to more lines of therapy and thus potentially increase OS.

Because mCRPC management increasingly combines systemic, metastasis-directed, and primary-directed treatments, accurate imaging-based response assessment will be needed to guide their sequencing [40]. Prospective studies are warranted to assess MET-RADS-P as a structured imaging tool in trials.

  ***Author contributions:*** Luca Russo had full access to all the data in the study and takes responsibility for the integrity of the data and the accuracy of the data analysis.

  *Study concept and design:* Tunariu.

*Acquisition of data:* Abramovicz, Avesani, Longoria, Russell-Rose.

*Analysis and interpretation of data:* Bottazzi, Longoria, Russo, Taha.

*Drafting of the manuscript:* Longoria, Withey.

*Critical revision of the manuscript for important intellectual content:* Tunariu, de Bono, Sala.

*Statistical analysis:* Porta, Biscombe.

*Obtaining funding:* Koh, Sharp, Tunariu.

*Administrative, technical, or material support:* Russell-Rose, Blackledge.

*Supervision:* Paschalis, D’Erme.

*Other:* None.

***Financial disclosures:***

Luca Russo certifies that all conflicts of interest, including specific financial interests and relationships and affiliations relevant to the subject matter or materials discussed in the manuscript (eg, employment/ affiliation, grants or funding, consultancies, honoraria, stock ownership or options, expert testimony, royalties, or patents filed, received, or pending), are the following: A.S. has received travel support from Sanofi, Roche Genentech and Nurix, and speaker honoraria from Astellas Pharma and Merck Sharp & Dohme. He has served as an advisor to DE Shaw Research, CHARM Therapeutics, Ellipses Pharma, and Droia Ventures. AS has been the Chief Investigator/Principal Investigator of industry-sponsored clinical trials.

A.P. has served as an advisor to Boehringer Ingelheim.

J.d.B. has served on advisory boards and received fees from many companies including AbbVie, Acai Therapeutics, Amgen, Amunix, Astellas, Bayer, Bioxcel Therapeutics, Boehringer Ingelheim, Celcuity, Daiichi, Dark Blue Therapeutics, Duke Street Bio Limited, Dunad Therapeutics, Eikon Therapeutics, Endeavor Biomedicines Inc., Genentech/Roche, GSK, Macrogenics, Merck Serono, MetaCurUm, Moma, Morphosys, Myricx, Novartis, Nurix Therapeutics, Nuvation Bio, Oncopia Therapeutics, Inc. d/b/a SK Life Science Labs, One-Carbon Therapeutics Inc, Oncternal, Orion, Page Therapeutics, Peptone, Pfizer, Takeda, Tango Therapeutics, Tubulis GmbH, VIR Biotechnology. He is an employee of the Institute of Cancer Research, which has received funding or other support for his research work from AstraZeneca, Crescendo, Immunic Therapeutics, MetaCurUm, Morphosys, Myricx, Nurix Therapeutics, Oncopia Therapeutics, Inc. d/b/a SK Life Science Labs, Oncternal, Orion, Sanofi Aventis. The ICR has a commercial interest in abiraterone, PARP inhibition in DNA repair defective cancers, and PI3K/AKT pathway inhibitors (no personal income). J.D.B. was named as an inventor, with no financial interest, for patent 8 822 438, submitted by Janssen that covers the use of abiraterone acetate with corticosteroids. He has been the CI/PI of many industry-sponsored clinical trials. J.D.B. is a National Institute for Health and Care Research (NIHR) senior investigator. The views expressed in this article are those of the authors and not necessarily those of the National Health System, the NIHR, or the Department of Health. The remaining authors have nothing to disclose.

  ***Funding/Support and role of the sponsor***: None.

  ***Acknowledgments:*** This work was supported by Prostate Cancer UK through a Research Innovation Awards (RIA-18-ST2-23).
