# Supplementary material for: Comparative assessment of METastasis Reporting and Data System for Prostate Cancer versus Prostate Cancer Clinical Trials Working Group 3 Criteria in assessing treatment response in patients with metastatic castration-resistant prostate cancer
Source: Eur Urol Open Sci. 2026 Aug 20;92:1–9. doi: 10.1016/j.euros.2026.08.003 (PMC13524628; doi:10.1016/j.euros.2026.08.003)
Supplement: Supplementary file 1 [file mmc1.docx]

**Supplementary material**

Supplementary Methods. Multivariable model specification

We assessed the prognostic value of early MET-RADS-P response (≤12 weeks) on overall survival using a multivariable Cox proportional-hazards model. We pre-specified the covariates from prognostic factors established in mCRPC. We entered all covariates simultaneously. We applied no stepwise or data-driven variable selection. This gave the adjusted effect of early MET-RADS-P response while accounting for known confounders. The model included: early MET-RADS-P response (≤12 weeks), the variable of interest; treatment type (corresponding to the imaging assessment) and number of previous treatment lines; age and time since diagnosis (years), modelled as continuous variables; Gleason score (≤7, 8, or ≥9); ECOG performance status (0 vs 1–2); opiate use; presence of nodal or visceral disease at baseline; baseline laboratory values, log-transformed: haemoglobin, platelet count, neutrophil-to-lymphocyte ratio, alkaline phosphatase, albumin, lactate dehydrogenase, and PSA. Adjusted hazard ratios with 95% confidence intervals for every covariate are reported in Supplementary Table 7.

**Supplementary Table 1.** MET-RADS-P assessment categories.

| **RAC** | **Classification** | **Region** | **Descriptions** |
| --- | --- | --- | --- |
| 1 | *Highly likely to be responding* | Local, nodal, and visceral | Consistent with RECIST v1.1/PCWG criteria for unequivocal response (partial/complete) |
|  |  | Bone | • Return of normal marrow in areas previously infiltrated by focal/diffuse metastatic infiltration  • Decrease in number/size of focal lesions sufficient to indicate high likelihood response |
|  |  |  | • Evolution of diffuse neoplastic pattern to focal lesions |
|  |  |  | • Decreasing soft tissue associated with bone disease |
|  |  |  | • Dense lesion sclerosis (edge to edge), sharply defined, very thin/disappearance of hyperintense rim on T2W-FS images |
|  |  |  | • The emergence of intra/peritumoural fat within/around lesions (fat dot/halo signs) |
|  |  |  | • Previously evident lesion shows increase in ADC from 1400 mm2/s to >1400 mm2/s |
|  |  |  | • 40% increase in ADC from baseline with corresponding decrease in high *b-*value SI; and morphological findings consistent with stable or responding disease |
| 2 | *Likely to be responding* | Local, nodal, and visceral | Changes depicting tumour response that do not meet RECIST v1.1/PCWG criteria for partial or complete response |
|  |  | Bone | Evidence of improvement, but not enough to fulfil criteria for RAC 1. For example: |
|  |  |  | • Previously evident lesions showing increases in ADC from 1000 mm2/s to <1400 mm2/s |
|  |  |  | • >25% but <40% increase in ADC from baseline with corresponding decrease in high *b-*value SI; and morphological findings consistent with stable or responding disease |
| 3 | *No change* | All | No observable change |
| 4 | *Likely to be progressing* | Local, nodal, and visceral | Changes depicting tumour progression that do not meet RECIST v1.1/PCWG criteria for and visceral progression |
|  |  | Bone | • Evidence of worsening disease, but not enough to fulfil criteria for RAC 5  • Equivocal appearance of new lesion(s) |
|  |  |  | • No change in size but increasing SI on high *b-*value images (with ADC values < 1400 mm2/s) consistent with possible disease progression |
|  |  |  | • Relapse disease: re-emergence of lesion(s) that previously disappeared or enlargement of lesion(s) lesions that had partially regressed/stabilized with prior treatments |
|  |  |  | • Imaging depicted bone lesions that might be clinically significant (therefore excludes asymptomatic fractures in noncritical bones) |
|  |  |  | • Soft tissue in spinal canal causing narrowing not associated with neurological findings and not requiring radiotherapy |
| 5 | *Highly likely to be progressing* | Local, nodal, and visceral | Changes depicting tumour progression that meet RECIST v1.1/PCWG criteria for unequivocal progression |
|  |  | Bone | • New critical fracture(s)/cord compression requiring radiotherapy/surgical intervention (**!** only if confirmed as malignant by MRI signal characteristics) |
|  |  |  | • Unequivocal new focal/diffuse area(s) of metastatic infiltration in regions of prior normal marrow  • Unequivocal increase in number/size of focal lesions |
|  |  |  | • Evolution of focal lesions to diffuse neoplastic pattern |
|  |  |  | • Appearance/increasing soft tissue associated with bone disease |
|  |  |  | • New lesions/regions of high signal intensity on high *b-*value images with ADC value between 600 mm^2^/s and 1000 mm^2^/s |

**Supplementary Table 2.** Systemic treatments administered during study period (n=210).

| **Treatment** | **N** | **%** |
| --- | --- | --- |
| **Total lines** | **210** | **100** |
| Androgen receptor pathway inhibitor (ARPI) | 6 | 2.86 |
| Antiangiogenic | 1 | 0.48 |
| Antibody-drug conjugates (ADC) | 8 | 3.81 |
| Chemokine/cytokine antagonists | 19 | 9.05 |
| Cytotoxic chemotherapy | 4 | 1.90 |
| Defective DNA repair-targeting agents | 53 | 25.24 |
| Epigenetic modulators | 14 | 6.67 |
| Immunotherapy | 60 | 28.57 |
| Intracellular signalling inhibitor | 10 | 4.76 |
| Microbiota-targeting trial | 2 | 0.95 |
| Radioligand therapy | 33 | 15.71 |

**Supplementary Table 3.** Overall response assessment according to MET-RADS-P and PCWG3 criteria. Concordant responses were highlighted in green. Agreement 301/467, 64.5% (95% CI: 59.8, 68.7). RAC: response assessment category.

| **OVERALL** | |  |  |  | **PCWG3** |  |  |  |
| --- | --- | --- | --- | --- | --- | --- | --- | --- |
|  |  | **No disease** | **CR** | **PR** | **SD** | **PD** | **PD-U** | **Total** |
| **MET-RADS-P** |  |  |  |  |  |  |  |  |
| **Responding (RAC 1-2)** | **N** | 0 | 2 | 39 | 34 | 0 | 0 | 75 |
|  | **%** | 0.0 | 0.4 | 8.3 | 7.3 | 0.0 | 0.0 | 16.1 |
| **Stable (RAC 3)** | **N** | 1 | 0 | 1 | 77 | 0 | 2 | 81 |
|  | **%** | 0.2 | 0.0 | 0.2 | 16.5 | 0.0 | 0.4 | 17.3 |
| **Progressing (RAC 4-5)** | **N** | 2 | 5 | 18 | 103 | 162 | 21 | 311 |
|  | **%** | 0.4 | 1.1 | 3.9 | 22.1 | 34.7 | 4.5 | 66.6 |
| **Total** | **N** | 3 | 7 | 58 | 214 | 162 | 23 | 467 |
|  | **%** | 0.6 | 1.5 | 12.4 | 45.8 | 34.7 | 5.0 | 100.0 |

**Supplementary Table 4.** Bone-only response assessment according to MET-RADS-P and PCWG3 criteria. Concordant responses were highlighted in green. Agreement: n=207/467, 44.4% (95%CI: 39.8, 48.9). RAC: response assessment category. For the PD-U instances (49) in which a BS confirmation was not obtained, 9 (12%) were patients in which treatment was discontinued due to clinical and PSDA progression and 40 ((55%) paired assessments were classified as PD on PCWG3 assessments due to PS in soft tissue disease by RECIST.

| **BONE** | |  | **PCWG3** |  |  |  |
| --- | --- | --- | --- | --- | --- | --- |
|  |  | **No disease** | **Non-PD** | **PD-U** | **PD** | **Total** |
| **MET-RADS-P** |  |  |  |  |  |  |
| **No disease** | N | 31 | 0 | 0 | 0 | 31 |
|  | % | 6.6 | 0.0 | 0.0 | 0 | 6.6 |
| **Responding (RAC 1-2)** | N | 2 | 63 | 1 | 0 | 66 |
|  | % | 0.4 | 13.5 | 0.2 | 0.0 | 14.1 |
| **Stable (RAC 3)** | N | 0 | 83 | 1 | 0 | 84 |
|  | % | 0.0 | 17.8 | 0.2 | 0 | 18.0 |
| **Progressing (RAC 4-5)** | N | 2 | 191 | 71 | 22 | 286 |
|  | % | 0.4 | 40.9 | 15.2 | 4.7 | 61.2 |
| **Total** | N | 35 | 337 | 73 | 22 | 467 |
|  | % | 7.5 | 72.2 | 15.6 | 4.7 | 100.0 |

.

**Supplementary Table 5.** Soft tissue-only response assessment according to MET-RADS-P and PCWG3 criteria. Concordant responses are highlighted. Agreement: n=415/467, 88.8% (95%CI: 85.7, 91.6). RAC: response assessment category.

| **RECIST** | | **PCWG3** | | | | | **Total** |
| --- | --- | --- | --- | --- | --- | --- | --- |
|  |  | **No disease** | **CR** | **PR** | **SD** | **PD** |  |
| **MET-RADS-P** |  |  |  |  |  |  |  |
| **No disease** | N | 88 | 0 | 0 | 1 | 0 | 89 |
|  | % | 19.0 | 0.0 | 0.0 | 0.2 | 0.0 | 19.1 |
| **CR** | N | 11 | 6 | 0 | 0 | 0 | 17 |
|  | % | 2.4 | 1.3 | 0.0 | 0.0 | 0.0 | 3.6 |
| **PR** | N | 6 | 0 | 61 | 0 | 0 | 67 |
|  | % | 1.3 | 0.0 | 13.1 | 0.0 | 0.0 | 14.4 |
| **SD** | N | 15 | 0 | 0 | 100 | 0 | 115 |
|  | % | 3.2 | 0.0 | 0.0 | 21.4 | 0.0 | 24.6 |
| **PD** | N | 6 | 0 | 3 | 10 | 160 | 179 |
|  | % | 1.3 | 0.0 | 0.7 | 2.1 | 34.3 | 38.3 |
| **Total** | N | 126 | 6 | 64 | 111 | 160 | 467 |
|  | % | 27.0 | 1.3 | 13.7 | 23.8 | 34.3 | 100.0 |

**Supplementary Table 6. Results from the cumulative logit mixed model – estimates of systematic and random differences between response assessment methods**

|  | **Overall disease** | | **Bone disease** | | **Soft disease** | |
| --- | --- | --- | --- | --- | --- | --- |
|  | **Est** | **95% Cr Int** | **Est** | **95% Cr Int** | **Est** | **95% Cr Int** |
| ***Assuming independence between scans*** |  |  |  |  |  |  |
| Diff METRADS- PCWG3* | +1.27 | 0.17-0.94 | +1.44 | 1.13-1.76 | +1.2 | 0.22-0.78 |
| Latent ICC | 0.664 | 0.580-0.738 | 0.614 | 0.525-0.693 | 0.958 | 0.943-0.970 |
| Probability agreement | 0.432 | 0.379-0.602 | 0.472 | 0.309-0.585 | 0.635 | 0.432-0.816 |
|  |  |  |  |  |  |  |
| ***Accounting for clustered data*** |  |  |  |  |  |  |
| Diff METRADS- PCWG3* | +1.05 | 0.15-0.75 | +1.49 | 0.17-1.17 | +0.58 | 0.15-0.29 |
| Latent ICC | 0.57 | 0.469-0.667 | 0.732 | 0.661-0.797 | 0.856 | 0.805-0.899 |
| Probability agreement | 0.39 | 0.350-0.474 | 0.434 | 0.331-0.545 | 0.629 | 0.441-0.815 |

**Systematic difference between methods; model parameter in the logit scale (>0 values indicate METRADS have higher scores).*

*Diff=difference; ICC: intraclass correlation coefficient; Est: estimate; 95% Cr Int= 95% credible interval. All estimates derived from the posterior probability.*

*A model assuming independence between scans within the same patient is also included for reference.*

**Supplementary Table 7.** Multivariate Cox model of overall survival based on WB-MRI ≤12 weeks (N=143 patients with complete data in all variables)

|  |  | **Univariable models** | | | | **Multivariable model** | | | |
| --- | --- | --- | --- | --- | --- | --- | --- | --- | --- |
| **Characteristic** | **Events/N** | **HR** | **p-value** | **95% CI** | | **HR** | **p-value** | **95% CI** | |
| **METRADS-P** |  |  |  |  |  |  |  |  |  |
| Responding | 14/17 | 1.00 | - |  |  | 1.00 | - |  |  |
| Stable | 25/33 | 0.96 | 0.896 | 0.59 - | 1.85 | 1.09 | 0.828 | 0.49 - | 2.44 |
| Progressing | 75/93 | 2.41 | 0.004 | 1.33 - | 4.37 | 4.75 | <0.001 | 2.12 - | 10.65 |
|  |  |  |  |  |  |  |  |  |  |
| **Age** | 114/143 | 1.02 | 0.223 | 0.99- | 1.04 | 1.07 | <0.001 | 1.03 - | 1.11 |
| **Gleason score** |  |  |  |  |  |  |  |  |  |
| ≤7 | 27/36 | 1.00 | - |  |  | 1.00 | - |  |  |
| 8 | 26/30 | 0.76 | 0.330 | 0.44 - | 1.31 | 1.09 | 0.805 | 0.55 - | 2.15 |
| 9 or 10 | 61/77 | 0.71 | 0.149 | 0.45 - | 1.13 | 0.89 | 0.698 | 0.48 - | 1.63 |
| **ECOG PS** |  |  |  |  |  |  |  |  |  |
| 0 | 15/22 | 1.00 | - |  |  | 1.00 | - |  |  |
| 1-2 | 99/121 | 2.26 | 0.007 | 1.25 - | 4.10 | 1.71 | 0.129 | 0.86 - | 3.41 |
| **Use of opiates** |  |  |  |  |  |  |  |  |  |
| No | 72/97 | 1.00 | - |  |  | 1.00 | - |  |  |
| Yes | 42/46 | 2.34 | <0.001 | 1.58 | 3.48 | 2.84 | <0.001 | 1.71 - | 4.74 |
|  |  |  |  |  |  |  |  |  |  |
| **Years since diagnosis** | 114/143 | 0.99 | 0.599 | 0.95 | 1.03 | 0.92 | 0.011 | 0.86 | 0.98 |
| **Haemoglobin (log)** | 114/143 | 0.19 | 0.037 | 0.04 - | 0.90 | 0.86 | 0.883 | 0.11 | 6.67 |
| **Platelets (log)** | 114/143 | 1.93 | 0.041 | 1.03 - | 3.63 | 1.67 | 0.183 | 0.79 - | 3.53 |
| **Neutrophil to lymphocyte ratio (log)** | 114/143 | 1.31 | 0.109 | 0.94 - | 1.81 | 0.77 | 0.260 | 0.49 - | 1.21 |
| **Alkaline phosphatase (log)** | 114/143 | 1.87 | <0.001 | 1.45- | 2.41 | 1.72 | 0.001 | 1.24 - | 2.38 |
| **Albumin (log)** | 114/143 | 1.13 | 0.846 | 0.33 - | 3.91 | 0.96 | 0.955 | 0.25 - | 3.65 |
| **Lactate dehydrogenase (log)** | 114/143 | 2.43 | <0.001 | 1.52 - | 3.88 | 0.84 | 0.668 | 0.38 - | 1.84 |
| **Prostate specific antigen (log)** | 114/143 | 1.15 | 0.008 | 1.04 - | 1.28 | 0.94 | 0.406 | 0.81 | 1.09 |
| **Nodal disease** |  |  |  |  |  |  |  |  |  |
| No | 63/77 | 1.00 | - |  |  | 1.00 | - |  |  |
| Yes | 51/66 | 1.03 | 0.869 | 0.71 - | 1.49 | 0.81 | 0.458 | 0.47 - | 1.41 |
| **Visceral disease** |  |  |  |  |  |  |  |  |  |
| No | 81/108 | 1.00 | - |  |  | 1.00 | - |  |  |
| Yes | 33/35 | 1.49 | 0.053 | 0.99 - | 2.24 | 1.68 | 0.062 | 0.97 - | 2.90 |
| **Prior lines of treatment** |  |  |  |  |  |  |  |  |  |
| ≤1 | 17/22 | 1.00 | - |  |  | 1.00 | - |  |  |
| 2 | 32/41 | 1.36 | 0.314 | 0.75 - | 2.50 | 1.17 | 0.693 | 0.54 - | 2.53 |
| 3 | 42/53 | 2.34 | 0.004 | 1.31 - | 4.18 | 3.08 | 0.006 | 1.39 - | 6.81 |
| 4+ | 23/27 | 2.85 | 0.002 | 1.48 - | 5.48 | 2.97 | 0.012 | 1.27 - | 6.92 |
| **Treatment** |  |  |  |  |  |  |  |  |  |
| Chemokine/cytokine antagonist | 11/12 | 1 |  |  |  | 1 |  |  |  |
| DDR-targeting drug | 34/36 | 0.85 | 0.639 | 0.43- | 1.69 | 1.47 | 0.398 | 0.60 - | 3.62 |
| Epigenetic regulator | 8/9 | 0.64 | 0.349 | 0.26- | 1.62 | 0.85 | 0.773 | 0.29 - | 2.53 |
| Immunotherapy | 27/43 | 1.00 | 0.995 | 0.49- | 2.04 | 1.20 | 0.670 | 0.52 - | 2.76 |
| Radioligand therapy | 21/22 | 1.41 | 0.363 | 0.67- | 2.93 | 1.16 | 0.742 | 0.48 - | 2.78 |
| Other | 13/21 | 1.17 | 0.709 | 0.52- | 2.64 | 1.77 | 0.253 | 0.67 - | 4.71 |

**Supplementary Figure 1.** Sankey diagram illustrating soft tissue-only assessment comparison between PCWG3 and MET-RADS-P criteria. Both PCWG3 (left) and MET-RADS-P (right) follow RECIST 1.1 for the assessment, and patients can be classified as No disease, CR (complete response), PR (partial response), SD (stable disease), and PD (progressive disease).
**Supplementary Figure 2.** A 64-year-old patient with *BRCA2*-mutated mCRPC, treated with a PARP inhibitor. PSA decreased from 89 µg/L at baseline, to 35 µg/L at Timepoint 1, and later to 28 µg/L. Bone scintigraphy showed additional lesions at Timepoint 1 (PD-U) but no additional lesions at Timepoint 2, consistent with a flare response. DWI at baseline showed a higher burden of metastatic disease than BS. The ADC values in a representative thoracic vertebral body metastasis increased from 840 to 1690 and then 2070 10-6mm2/s, consistent with decreasing cellularity and a favourable response. The MET-RADS-P RAC was 1 (highly likely to be responding).

**Supplementary Figure 3.** A 72-year-old patient with *BRCA2*-mutated mCRPC, treated with a PARP inhibitor. PSA increased from 16 µg/L at baseline, to 22 and then 40 µg/L. On bone scintigraphy, several new lesions were seen at Timepoint 1 (PD-U), with many additional lesions at Timepoint 2 (PD). Contemporaneous DWI demonstrated many new and enlarging metastases at Timepoint 1 (MET-RADS-P RAC 5, highly likely to be progressing), with further significant increase in disease burden by Timepoint 2, 8 weeks later. This case highlights how disease burden can be under-represented on conventional imaging and can markedly increase whilst awaiting the confirmatory imaging required by PCWG3 criteria.
